# A HRM Real-Time PCR Assay for Rapid and Specific Identification of the Emerging Pest Spotted-Wing Drosophila (*Drosophila suzukii*)

**DOI:** 10.1371/journal.pone.0098934

**Published:** 2014-06-13

**Authors:** Manpreet K. Dhami, Lalith Kumarasinghe

**Affiliations:** Plant Health and Environment Laboratory, Ministry for Primary Industries, Auckland, New Zealand; Naval Research Laboratory, United States of America

## Abstract

Spotted wing drosophila (*Drosophila suzukii*) is an emerging pest that began spreading in 2008 and its distribution now includes 13 countries across two continents. Countries where it is established have reported significant economic losses of fresh produce, such as cherries due to this species of fly. At larval stages, it is impossible to identify due to its striking similarities with other cosmopolitan and harmless drosophilids. Molecular methods allow identification but the current technique of DNA barcoding is time consuming. We developed and validated a rapid, highly sensitive and specific assay based on real-time PCR and high resolution melt (HRM) analysis using EvaGreen DNA intercalating dye chemistry. Performance characteristics of this qualitative assay, validation and applicability in a New Zealand quarantine framework are discussed. Application of this robust and independently validated assay across the spectrum of key food production and border protection industries will allow us to reduce the further spread of this damaging species worldwide.

## Introduction

The first step for the effective management of a pest is its reliable and rapid identification. Rapid identification techniques are not often available for emerging pests. Such is the case with the spotted-wing drosophila, *Drosophila suzukii* (Diptera: Drosophilidae). *Drosophila suzukii* is a pest of major concern across the world, both in countries where the fly is established and countries where it is currently not present [1]. It has been placed on the European and Mediterranean Plant Protection Organisation (EPPO) Alert List, as it has a “high potential for spread and can cause economic damage to many fruit crops” [1]. For example, in 2009, US$ 2.6 billion worth of strawberry, blueberry and cherry production was lost due to damage by *D. suzukii* in California, Washington and Oregon alone [2].

*Drosophila suzukii* is considered native to South East Asia, possibly originating in South China – Northern India region and spreading across to Japan in the early 1900s [3]. In the 1980s, it was reported from Hawaii but not considered a pest [4]. It has expanded its range considerably since 2008, when it was first misidentified in a Santa Cruz County raspberry field in California, USA [5]. Around the same time, it was also reported from Spain [6]. By 2009, it was found across 20 counties in California and had spread to Oregon, Washington and Florida in the USA and British Columbia in Canada [5]. From 2010 to 2013 it has continued to spread across northern America and mainland Europe [7]. It is not present in Mexico, Italy, France, Switzerland, Slovenia, Croatia, Austria, Germany, Belgium and the United Kingdom, in addition to parts of USA, Canada and Spain [1],[7]–[9].

Climate modelling studies predict that this species will continue to spread all across the USA and continental Europe, including even the Scandinavian countries in some scenarios [7], [10]. Several factors suggest that this species has the potential to become a global problem for fruit production [7]. Unlike most drosophilids, the females of this species are able to lay eggs in healthy, unwounded fruits, [11]. High fecundity [3], wide host range [12], resistance to parasitoids [13], high dispersal potential, aided with tolerance for a wide range of climatic conditions [14] demonstrates its pest potential. Several countries outside its current distribution, such as New Zealand and Australia, recognise *D. suzukii* as an unwanted species and impose strict border controls to reduce the risk of invasion [15], [16].

A large proportion of suspected drosophilids in transit or discovered in orchard surveys are in the larval stages, as their feeding leads to observable damage to the produce [15]. As discussed above, it is nearly impossible to identify them accurately, unless they are reared to adults. This can be a high risk task, as many facilities around the world may not have the quarantine security required for rearing pests such as *D. suzukii*, and failure rate of rearing from eggs can be very high [17]. Additionally, the lengthy time component of rearing to identification may be frustrating when hundreds of thousands to millions of dollars worth of fresh produce is at stake.

In such circumstances, molecular identification techniques can provide the solution. Polymerase chain reaction (PCR)-based methods have been used for several decades now for the identification of pests and diseases all around the world [18]–[21]. Techniques such as DNA barcoding, restriction fragment length polymorphism and microsatellite analysis allow for the identification of otherwise difficult to identify species. DNA barcoding has been developed for *D. suzukii* and is currently the only published molecular technique available for its accurate identification [5]. Several sequences of *D. suzukii* COI (cytochrome oxidase I) genes are available in GenBank [22] and BOLD [23] databases. All of these PCR-based methods are however, open tube-based, *i.e.* they require post processing of the PCR product, such as gel electrophoresis analysis or sequencing of the product. Open-tube methods have an increased chance of contamination. Such methods may also include multiple post-reaction processing steps, such as restriction digests, which would require significant resourcing if hundreds of samples were involved. With the advent of fluorescence-based real-time PCR [24], these post-reaction processes are eliminated and the closed-tube approach also minimises the potential for contamination. It is often more sensitive and accurate in identifying target organisms compared to other DNA-based methods [25], and is independent of the life stage involved.

*Drosophila suzukii* belongs to the genus *Sophophora* and forms its own species subgroup under the *melanogaster* species group [26]. There are detailed morphological identification keys available for a wide range of species from this group, and recently, a refined identification guide for *D. suzukii* and closely related species was published [5]. Unfortunately, like most insects, species-level identification keys are available only for adults and identification of larvae, pupae and eggs is guesswork at best. There are 15 closely related species in the *D. suzukii* subgroup and their interspecies relationships are yet to be fully resolved [27]. A recent phylogenetic analysis based on 17 gene regions, established the long-debated monophyly of the *D. suzukii* species subgroup [26]. In this analysis, among other gene regions, COI was able to be used to differentiate between various species of this subgroup.

In this study, we developed a highly specific and sensitive real-time PCR approach to accurately identify *D. suzukii* samples. Real-time PCR is based on two major chemistries, the fluorescence probe-based chemistry and the DNA-binding dye-based chemistry [28]. We selected the DNA-binding dye-based chemistry as it is simple [29], and requires non-fluorescent oligos that can be cheaply synthesised. This real-time PCR approach can easily be coupled with high-resolution melt (HRM) analysis, which can provide single base-pair differentiation amongst target and non-target DNA [30]. We used the COI gene as the target, as it is well characterised for a range of species from the *Drosophila melanogaster* group, including sequences available for some species from the *D. suzukii* species subgroup. We report here the developed assay and its performance criteria, such as analytical and diagnostic specificity, analytical sensitivity, repeatability, reproducibility and blind panel testing. The validation and application of the assay within a quarantine framework is discussed.

## Experimental Methods

### Sample collection and identification

Three main types of drosophilids were examined in this study: Target species: *D. suzukii* samples; Non-target closely related species: *D. suzukii* species subgroup and sister group species; and Non-target New Zealand (NZ) drosophilids: native and introduced drosophilids present in New Zealand. New Zealand does not have *D. suzukii*, therefore voucher specimens were sourced from a range of recognized researchers from reference laboratories across the United States and continental Europe. The samples had either been captured in the wild or from laboratory colonies. Some samples of *D. suzukii* (from Japan) and of closely related species belonging to *D. suzukii* species subgroup were purchased from the laboratory colonies maintained by the Drosophila Species Stock Centre, UC San Diego, USA.

Freshly killed flies were preserved in 1.5 mL tubes with a small amount of Ethanol (95% - absolute) or RNA*later* (Invitrogen, Carlsbad, CA, USA) for shipment and were kept in their original preservatives for storage post transit. Non-target NZ species were either freshly collected by Dr. Simon Hodge (Lincoln University, New Zealand), or available in the Plant Health and Environment Laboratory (PHEL) Ethanol collection. Expert identifications of the flies were sought from sample submitters and identities of the samples were confirmed by entomologist Dr. Disna Gunawardana (PHEL). No specific permits were required for sample collections and sample submitters listed should be contacted for individual sampling details. No samples were collected in national parks and collection permits were not required (exception: some samples for colony rearing by DSSC, which are covered by their respective permits). No endangered or threatened flies were included in this study. All flies imported into New Zealand were in accordance to the Import Health Standard, Section 22 of the Biosecurity Act 1993. Ethics approval was not required as insects are not classified as animals for the purposes of the Animal Welfare Act, 1999, New Zealand Legislation.

### DNA extraction, PCR amplification and sequencing

DNA was extracted using DNeasy Blood and Tissue kit (Qiagen, Valencia, CA, USA) as per the manufacturer’s instructions. Since drosophilids are very small in size, physical disruption of tissue was performed by finely chopping with sterile scissors. For some samples, the enzymatic prepGem DNA prep kit (ZyGem Corporation Ltd., Hamilton, New Zealand) was used to extract DNA, as per the manufacturer’s instructions. This is a rapid method of DNA extraction and provides a time advantage, especially for urgent diagnostic needs. DNA extracts were quantified on a NanoDrop 3300 spectrophotometer (Thermo Fisher Scientific Inc., Wilmington, DE, USA).

Molecular identification of all the samples used in the assay development was also conducted by PCR amplification and sequencing of the COI gene region. Universal insect COI primer pair LCO1490 and HCO2198 [31] was used for the amplification of approximately 700 bp region. Each 20 µL reaction consisted of 1 × Red N’Amp master mix (Sigma-Aldrich Co., St. Louis, MO, USA), 250 nM of each primer, 0.04 µg/μL Bovine Serum Albumin (BSA) (Sigma-Aldrich Co.), 2–5 ng of DNA template and PCR-grade water. Cycling conditions were: initial denaturation at 94°C for 2 min, 30 cycles of 94°C for 15 sec, 52°C for 30 sec and 72°C for 45 sec, followed by final extension step of 7 min at 72°C. The amplicons were electrophoresed on 1% TAE-agarose gel stained with SYBR safe, and observed under UV illumination using the Gel-Doc system (BioRad, Hercules, CA, USA) and images processed using the Quantity One 1-D analysis software (BioRad). Successfully amplified products were sequenced bi-directionally using the amplification primers, by EcoGene (Auckland, New Zealand). All sequences were edited in Geneious Pro 5.5.6 (Biomatters Ltd, Auckland, New Zealand). Sequences were blasted against the GenBank nr database [32] or BOLD [23] database to confirm morphological identification. These sequences were used in the assay design. Sequences have been submitted to GenBank and accession numbers are provided in Table S1.

### Assay design and SNP description

Cytochrome oxidase subunit I (COI) gene sequences of Drosophilidae, especially of the species belonging to the *D. suzukii* species subgroup, were obtained from samples described above, and downloaded from GenBank and BOLD sequence databases. A total of 87 sequences were aligned using the in-built Geneious aligner and the alignment was trimmed to obtain a total of 608 bp of each sequence aligned. The resultant alignment has been submitted to DRYAD data repository under the doi: 10.5061/dryad.h08b.

*Drosophila suzukii* sequences had a range of mismatches against the sequences of most other drosophilid species, with the exception of the congener, *D. subpulchrella*. Between *D. suzukii* and *D. subpulchrella* there was a difference of only two single nucleotide polymorphisms (SNPs) along the length of their COI sequences that could reliably separate the two species. High resolution melt (HRM) enables differentiation of SNPs, so this approach was incorporated in the assay design. Primers were designed using the Primer 3 plugin [33] in Geneious, putting constrains on amplicon size to <150bp and Tm between 55–65°C, and considering each SNP separately. The amplicon secondary structure at the primer annealing temperature was calculated using the mFold web server [34].

### Real-time PCR optimisation

A real-time PCR protocol was setup using *D. suzukii* positive control samples as well as several non-target species samples as negative controls. The designed primer pairs that amplify *D. suzukii* were used in a real-time PCR run on a CFX96 Touch Real-time platform (BioRad, Hercules, CA, USA). Optimisation gradients of temperature (55–65°C), primer concentration (50 nM–400 nM) and Mg^2+^ concentration (3 mM–5 mM) were run to optimise the PCR conditions using the SsoFast EvaGreen Supermix (BioRad). The assay was also preliminarily tested using the Accumelt HRM Supermix (Quanta Biosciences, Gaithersburg, MD, USA) which employs the SYTO9 green fluorescent dye and Platinum Quantitative PCR SYBR Supermix UDG (Invitrogen), which employs the SYBR Green I dye. The performance of the optimised assay on these mastermixes was compared to select the optimal mastermix.

For each reaction, 96-well clear bottom plates were used and all samples, standards and controls were run in duplicate wells. Cycling protocol including the HRM protocol was developed based on the optimised conditions. All fluorescent data was acquired to the SYBR channel at the end of each cycle. The amplification and melt curves were visualised using CFX Manager software v. 3.0 (BioRad). The difference curves for HRM were created and analysed using the Precision Melt Analysis software v. 1.2 (BioRad).

### Analytical and diagnostic specificity of the Real-time PCR assay for *D. suzukii*

We included a total of 65 voucher specimens in this assay of which 23 were target and the remaining non-target species. The analytical specificity is the percentage of samples of known identity of the target species that return a positive outcome in the assay while the diagnostic specificity is the percentage of non-target samples of known identity that return a negative outcome in the assay. All available samples were used to calculate analytical and diagnostic specificity for the identification of *D. suzukii*. All the samples used were identified prior to testing by conventional PCR and sequencing as described in section 2.2.

### Analytical sensitivity evaluation, amplification efficiency, repeatability and reproducibility of the Real-time PCR assay for *D. suzukii*

To evaluate the analytical sensitivity of the Real-time PCR method, the 714 bp template COI gene region, amplified as described in section 2.2 was used to prepare plasmid standards of known copy number. The amplicon was cloned using the TOPO TA vector Cloning kit (Invitrogen, Carlsbad, CA, USA) as per the manufacturer’s instructions. Cloning was performed for two biological samples of *D. suzukii*, DQ19 (Washington, USA) and DQ43 (Trentino, Italy) and for each of these samples, two clones each, containing the correct insert were selected for preparing standards.

Plasmid DNA was extracted using the Wizard Plus SV Miniprep (Promega, Madison, WI, USA) and quantified using MultiSkan GO DNA quantification system using a μDrop* plate (Thermo Fisher Scientific, Waltham, MA, USA) as per manufacturer’s instructions. The plasmid was digested with *EcoR I* to linearize it. Copy number was calculated using the following equation:
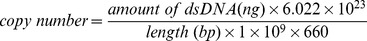



A dilution series of the plasmid from 10^7^−10^−1^ copies was created using the genomic DNA (Qiagen extraction) of a non-target fly, *Bactrocera invadens*.

Analytical sensitivity of the real-time PCR assay was determined using the dilution series with each concentration in quadruplicate. A smaller dilution series (10^−1^−10^−2^) of positively identified (by COI sequence and morphology) *D. suzukii* samples were run in parallel, to reflect a diagnostic situation. The amplification curves were fitted using the sigmoidal model with baseline correction using 1–12 cycles with the qpcR package [35] in the R environment [36]. Standard curves were built using the “calib” function with a fixed threshold of 200 and with 95% confidence values plotted. Efficiency was calculated by the qpcR package using the formula, 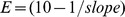. This was converted to percentage efficiency by using the formula, 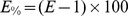. Fit of the slope was recorded as *r^2^* and the AIC statistic represented the fit of the model. Performance indicators such as the linear dynamic range and limit of detection were also calculated using the standard curve data [37].

Repeatability (intrarun variation) and reproducibility (interrun variation) for the real-time PCR assay were reported by means of *Cq* standard deviation and percent coefficient of variance (%CV) within and between runs, as this assay is non-quantitative. For repeatability we tested four samples (DQ11, DQ19, DQ20 and DQ23) in triplicate, in two identical runs and calculated the %CV for individual samples per run. This procedure, including two separate identical runs was performed at another Ministry for Primary Industry facility, the Animal Health Laboratory (AHL) to address repeatability externally. The data from all the runs was compiled to calculate %CV as a measure of reproducibility.

### Assay robustness: testing using old, degraded, low purity or low copy number samples

Fourteen samples of *D. suzukii* including single eggs, legs and dried material were extracted using prepGem DNA extraction kit (ZyGem Corporation Ltd., Hamilton, New Zealand). Individual eggs were punctured with a sterile fine needle before extraction and dried samples were soaked in the buffer for an extended time period (up to 1 hour), before extraction, following the manufacturer’s instructions. These samples were analysed using the real-time assay developed here to test for robustness and the ability to amplify degraded samples. All samples were run in duplicate wells, and positive and no template controls were included. These samples were also run using conventional PCR as described in section 2.2 for comparison.

### Assay validation: blind panel testing

A total of 23 samples of drosophilids were blinded to the tester by Dr. Disna Gunawardana (PHEL). These samples constituted a range of species, life stages, and body parts, mostly collected from interceptions in routine border or post-border surveys. DNA was extracted and quantified as described in section 2.2, followed by testing using the developed *D. suzukii* real-time assay. All samples were tested in duplicate and positive controls, negative controls and no template controls were used. The samples were also tested with a TaqMan 18S internal control real-time PCR (Applied Biosystems, CA, USA), as per manufacturer’s instructions to test for false negatives due to lack of DNA amplification. Furthermore, the samples were re-randomised, blinded and sent to the Animal Health Laboratory, Wellington for the independent external validation of the assay. These experiments were essentially a simulation of the New Zealand quarantine framework, in which the developed assay was applied.

## Results

### *Drosophila suzukii* assay: Identification via real-time PCR and high resolution melt analysis

**Figure 1:**
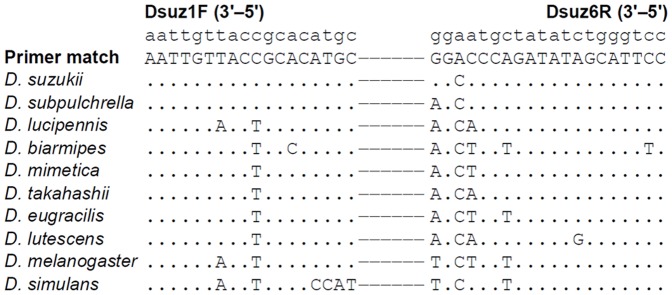
Real-time PCR assay for the identification of *Drosophila suzukii* primer pair alignment with closely related species. There is a mismatch in the reverse primer target sequence match at the 3′ end and non-target sequence *D. subpulchrella* (G → A), due to SNP-1. An additional mismatch was introduced in the reverse primer at third base from the 3′ end (A → C) to further suppress the binding of *D. subpulchrella*.

A SNP located at 186 bp position of the alignment (G →A) was the chosen site for the development of assay primers for *D. suzukii*. Primer pair, Dsuz1F (5′–AATTGTTACCGCACATGC–3′) and Dsuz6R (5′–GGAATGCTATATCTGGGTCC–3′), containing the SNP, was selected after *in silico* specificity testing against the sequence alignment using Geneious (Figure 1). The final alignment contained 32 *D. suzukii* and 8 *D. subpulchrella* sequences (DRYAD doi: 10.5061/dryad.h08b).

The assay was developed using EvaGreen DNA binding dye (in SsoFast mastermix formulation). Preliminary testing of Accumelt and SYBR Supermix for this assay revealed performance inferior to that of SsoFast mastermix (data not shown). The final reaction mixture and assay conditions are provided in Table 1.

**Table 1:** Qualitative real time PCR assay for the identification of *Drosophila suzukii*: Reaction composition and temperature cycling conditions.

Reaction composition		Cycling conditions			
Component	Final concentration	Step	Temperature	Time	
SsoFastTM Eva Green Mastermix	1×	Initial denature	95°C	2 min	
Primer F (Dsuz1F)	300 nm	Denature	95°C	10 sec	
Primer R (Dsuz6R)	300 nm	Anneal	61°C	40 sec	× 32 cycles
BSA	0.5 µg/μL	Extension*	72°C	20 sec	
DNA template	1–20 ng	Hold 1	95°C	10 sec	
PCR grade water	adjust volume to 10 µL	Hold 2	70°C	10 sec	
		Melting profile*	70°C–80°C		
			ramp speed: 0.2°C for 2 sec		

The denature, anneal and extension steps were cycled 32 times.

*Fluorescence signal was read in the SYBR channel at the end of each cycle during the cycling phase of the assay, and throughout the melting profile step, as identified by an asterisk.

**Figure 2:**
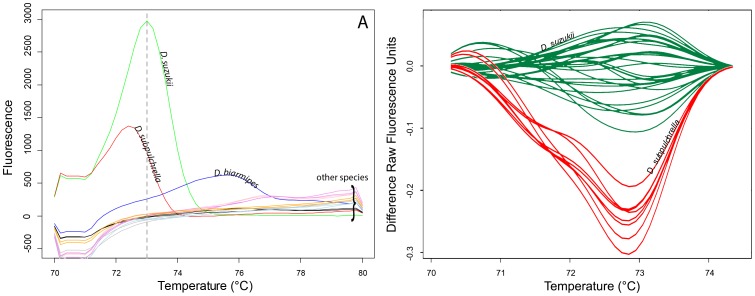
Species differentiation using the melting profile and high resolution melt analysis. A, Melting profiles of the species amplified by the *D. suzukii* assay primer pair Dsuz1F – Dsuz6R. Green  =  *D. suzukii*, Red  =  *D. subpulchrella* and Blue  =  *D. biarmipes*, other species did not amplify and had flat melt curves. B, Melt curve difference plots comparing Green  =  *D. suzukii* and Red  =  *D. subpulchrella* melt curves.

The selected primer pair preferentially amplified the target species *D. suzukii*, while non-target amplification of *D. subpulchrella* was observed on occasion at higher *Cq* (>26) and once for *D. biarmipes* after the *Cq* cut-off of 32 cycles. The primer pair amplified 117 bp of the target sequence and mFold indicated very little folding of this amplicon at 61°C (annealing temperature), with a low ΔG of 0.55 kcal/mol. The amplicon subjected to high resolution melt was able to distinguish between the two species based on the melting curve shape and melt peaks. The melt peak of the non-target species *D. subpulchrella* centred at 72.4°C (±0.2°C), while that of the target species *D. suzukii* centred at 73°C (−0.2°C/+0.4°C), based on a range of samples assessed over 10 runs. This range of melt peaks observed for *D. suzukii* was attributed to the presence of sequence variants from different populations, and therefore multiple positive controls, namely HRM1 (peak @ 73°C), HRM2 (peak @ 72.8°C) and HRM3 (peak @ 73.2°C) were used in diagnostic and blind panel assays, to pin point the sequence variants of *D. suzukii*. In most cases, the real-time component of the SYBR assay would suffice in the identification of *D. suzukii*, with the appropriate melt peaks. However, in the rare scenario that *D. subpulchrella* is encountered a comparison of the melt peaks and shape of the difference curves using high resolution melt curve analysis separates the two species clearly (Figure 2).

### Analytical and diagnostic specificity of the real-time PCR assay for *D. suzukii*: the use of HRM to distinguish between *D. suzukii* and *D. subpulchrella*

We were able to source five species belonging to the *D. suzukii* species subgroup, namely *D. suzukii suzukii* (multiple populations from Japan, Italy and USA), *D. lucipennis, D. biarmipes, D. mimetica* and *D. subpulchrella* (from Japan and China) (Table 2). We also obtained *D. takahashii, D. lutescens* and *D. eugracilis* that belong to sister groups of *D. suzukii* species subgroup (Table 2). There are 11 species of *Drosophila* present in New Zealand, of which we were able to source samples of nine species, namely *D. busckii, D. immigrans, D. melanogaster, D. simulans, D. hydei, D. repleta, D. kirki, D. neozealandica* and *D. pseudoobscura* (Table 2). Altogether we included 65 samples in this assay of which 23 were target and the remaining non-target species.

**Table 2:** Sample details.

Species	Population ID	*n*	Origin	Wild caught (WC)/colony(C)	Source/Collector^1^	Year (colony seed year)	Host (if known)	Morphology identifier	Preservation	DNA extraction method
***Drosophila suzukii*** ** species subgroup**										
*D. suzukii*	DS_US1	6	Oregon, USA	C	DS	2012	N/A	DS, DG	RNALater	QG
	DS_US2	4	Wapata, USA	C	PB	2012	N/A	PB, DG	95% Ethanol	QG
	DS_IT1	4	Trento, Trentino Italy	C	PB	2012	N/A	PB, DG	95% Ethanol	QG
	DS_IT2	4	Vizalgano, Trentino, Italy	WC	AG	2012	apple cider vinegar trap	AG, DG	70% Ethanol	QG
	DS_IT3	4	Bologna, Italy	WC	TR	2012	plum	TR, DG	95% Ethanol	QG
	DS_JP	2	Hachijo Island, Tokyo, Japan	C	DSSC	2012 (1978)	N/A	YF, DG	95% Ethanol	QG(1)/PG(1)
*D. subpulchrella*	DP_CH	7	China	C	DSSC	2012 (1991)	N/A	MW, DG	95% Ethanol	QG(6)/PG(1)
	DP_JP	4	Kanagawa, Japan	C	DSSC	2012 (1979)	N/A	MW, DG	95% Ethanol	PG(4)
*D. biarmipes*	DB_IN	2	Mysore, India	C	DSSC	2012 (1971)	N/A	AK, DG	95% Ethanol	QG(1)/PG(1)
*D. lucipennis*	DL_TW	2	Wulai, Taiwan	C	DSSC	2012 (1968)	N/A	LT, DG	95% Ethanol	QG(1)/PG(1)
*D. mimetica*	DM_BR	2	Ulu Temburong National Park, Kuala Belalong, Brunei	C	DSSC	2012 (2003)	N/A	AK, DG	95% Ethanol	QG(1)/PG(1)
**Sister groups to ** ***Drosophila suzukii*** ** species subgroup**										
*D. takahashii*	DT_TW	2	Yun Shui, Taiwan	C	DSSC	2012 (1968)	N/A	AK, DG	95% Ethanol	QG(1)/PG(1)
*D. lutescens*	DU_JP	2	Mito, Honshu, Japan	C	DSSC	2012 (1976)	N/A	DSSC, DG	95% Ethanol	QG(1)/PG(1)
*D. eugracilis*	DE_BR	2	Ulu Temburong National Park, Kuala Belalong, Brunei	C	DSSC	2012 (2003)	N/A	AK, OB, DG	95% Ethanol	QG(1)/PG(1)
**Common Drosophilidae/Native New Zealand Drosophilidae**										
*D. immigrans*	DI_NZ	5	Auckland, NZ	WC	Fruit fly survey	2011	methyl eugenol trap	DG	dry	QG
*D. pseudoobscura*	DO_NZ	4	New Brighton, MC, NZ	C	SH	2012	N/A	SH, DG	95% Ethanol	QG
*D. hydei*	DH_NZ	2	New Brighton, MC, NZ	WC	SH	2012	N/A	SH, DG	95% Ethanol	QG
*D. busckii*	DX_NZ	1	New Brighton, MC, NZ	WC	SH	2012	mushroom baits	SH, DG	95% Ethanol	QG
*D. simulans*	DC_NZ	1	New Brighton, MC, NZ	WC	SH	2012	N/A	SH, DG	95% Ethanol	QG
*D. melanogaster*	T13-444	1	**Thailand**	WC	PHEL border submission	2013	Capsicum	SH, DG	95% Ethanol	QG
*D. repleta*	DR_NZ	1	Auckland, NZ	WC	RHK	2009	N/A	DG	dry	QG
*D. neozealandica*	DN_NZ	1	Whangarei, ND, NZ	WC	JM Cawley	1997	*Zanthedeschia* spp. bulb	JC, DG	95% Ethanol	QG
*D. kirki*	DK_NZ	1	Oratia, AKL, NZ	WC	PHEL	1987	N/A (?)	DG	95% Ethanol	QG

The species, populations, biological replicates or no. of individuals tested (*n*), country of origin, type of source (wild/colony), source or collector, year of collection (colony seed year, if different), and host details are provided. All samples were morphologically identified by the collector or submitting organisation, and/or Dr. Disna Gunawardana (DG). Most samples were extracted using Qiagen (QG), while some were extracted using prepGem (PG), as described. For New Zealand locations, Crosby codes are used where needed to denote geographic area.

1 Identifiers and submitters: DS  =  David Shearer, Oregon State University, Oregon, USA; DG  =  Disna Gunawardana, Plant Health and Environment Laboratory (PHEL), Auckland, NZ; PB  =  Paul Becher, Swedish University of Agricultural Sciences, Alnarp, Sweden; AG  =  Alberto Grassi, Fondazione Edmund Mach/IASMA, Pergine Valsugana, Italy; TR  =  Tiso Roccini, Italan Plant Protection Service, Italy; DSSC  =  Drosophila Species Stock Centre, University of California, California, USA; YF  =  Yoshiyaki Fuyama, Tokya Metropolitan University, Japan; MW  =  Masayoshi Watada, Ehime University, Japan; AK  =  Artyom Kopp, University of California, Davis, USA; LT  =  Lynn Thockmorton, University of Chicago, USA; OB  =  Olga Barmina, University of California, Davis, USA; SH  =  Simon Hodge, University of Lincoln, Lincoln, NZ; JC  =  JM Cawley, PHEL submitter, RHK  =  Ruud H. Klienpaste, New Zealand.

#### SYBR specificity

All the 23 *D. suzukii* samples and an additional 7 *D. subpulchrella* samples amplified within the *Cq* cut-off of 32 cycles (Table 3). A single *D. biarmipes* sample showed amplification after the *Cq* cut-off. None of the remaining 34 non-target samples showed amplification.

**Table 3:** Specificity of the real-time PCR assay for the detection of *D. suzukii*.

S. no.	Sample ID	Real-time assay			Species^1^	Collection location	Year of collection	Life stage, body part (no. of individuals)	Preservation	DNA extraction method
		Result	Mean Cq	Mean Melt Peak (°C)						
1	DQ1	−	N/A	N/A	*D. immigrans*	Auckland, NZ	2011	adult, whole	dry	Qiagen
2	DQ2	−	N/A	N/A	*D. immigrans*	Auckland, NZ	2011	adult, whole	dry	Qiagen
3	DQ3	−	N/A	N/A	*D. immigrans*	Auckland, NZ	2011	adult, whole	dry	Qiagen
4	DQ4	−	N/A	N/A	*D. immigrans*	Auckland, NZ	2011	adult, whole	dry	Qiagen
5	DQ5	−	N/A	N/A	*D. immigrans*	Auckland, NZ	2011	adult, whole	dry	Qiagen
**6**	**DQ11**	**+**	**21.85**	**73**	***D. suzukii***	**Hachijo Is, Tokyo, Japan**	**2012**	**adult, whole**	**95% ethanol**	Qiagen
7	DQ12	−	33.47§	N/A	*D. biarmipes*	Mysore, India	2012	adult, whole	95% ethanol	Qiagen
8	DQ13	−	N/A	N/A	*D. lucipennis*	Wulai, Taiwan	2012	adult, whole	95% ethanol	Qiagen
**9**	DQ14	−	N/A	N/A	*D. mimetica*	Kuala Belalong, Brunei	2012	adult, whole	95% ethanol	Qiagen
10	DQ15	−	28.815	72.4	*D. subpulchrella*	China	2012	adult, whole	95% ethanol	Qiagen
11	DQ16	−	N/A	N/A	*D. takahashii*	Yun Shui, Taiwan	2012	adult, whole	95% ethanol	Qiagen
12	DQ17	−	N/A	N/A	*D. lutescens*	Mito, Honshu, Japan	2012	adult, whole	95% ethanol	Qiagen
13	DQ18	−	N/A	N/A	*D. eugracilis*	Kuala Belalong, Brunei	2012	adult, whole	95% ethanol	Qiagen
**14**	**DQ19**	**+**	**21.975**	**73.2**	***D. suzukii***	**Wapata, USA**	**2012**	**adult, whole**	**95% ethanol**	Qiagen
**15**	**DQ20**	**+**	**21.73**	**73.2**	***D. suzukii***	**Wapata, USA**	**2012**	**adult, whole**	**95% ethanol**	Qiagen
**16**	**DQ21**	**+**	**22.065**	**73.2**	***D. suzukii***	**Wapata, USA**	**2012**	**adult, whole**	**95% ethanol**	Qiagen
**17**	**DQ22**	**+**	**21.785**	**73.2**	***D. suzukii***	**Wapata, USA**	**2012**	**adult, whole**	**95% ethanol**	Qiagen
**18**	**DQ23**	**+**	**21.61**	**73**	***D. suzukii***	**Trento, Trentino, Italy**	**2012**	**adult, whole**	**95% ethanol**	Qiagen
**19**	**DQ24**	**+**	**21.815**	**73**	***D. suzukii***	**Trento, Trentino, Italy**	**2012**	**adult, whole**	**95% ethanol**	Qiagen
**20**	**DQ25**	**+**	**21.94**	**73**	***D. suzukii***	**Trento, Trentino, Italy**	**2012**	**adult, whole**	**95% ethanol**	Qiagen
**21**	**DQ26**	**+**	**22.35**	**73**	***D. suzukii***	**Trento, Trentino, Italy**	**2012**	**adult, whole**	**95% ethanol**	Qiagen
**22**	**DQ27**	**+**	**22.31**	**73**	***D. suzukii***	**Vizalgano, Trentino, Italy**	**2012**	**adult, whole**	**95% ethanol**	Qiagen
**23**	**DQ28**	**+**	**22.01**	**73**	***D. suzukii***	**Vizalgano, Trentino, Italy**	**2012**	**adult, whole**	**95% ethanol**	Qiagen
**24**	**DQ29**	**+**	**20.86**	**73**	***D. suzukii***	**Vizalgano, Trentino, Italy**	**2012**	**adult, whole**	**95% ethanol**	Qiagen
**25**	**DQ30**	**+**	**22.025**	**73**	***D. suzukii***	**Vizalgano, Trentino, Italy**	**2012**	**adult, whole**	**95% ethanol**	Qiagen
**26**	**DQ31**	**+**	**23.03**	**72.9**	***D. suzukii***	**Bologna, Italy**	**2012**	**adult, whole**	**95% ethanol**	Qiagen
**27**	**DQ32**	**+**	**22.585**	**73**	***D. suzukii***	**Bologna, Italy**	**2012**	**adult, whole**	**95% ethanol**	Qiagen
**28**	**DQ33**	**+**	**22.115**	**72.9**	***D. suzukii***	**Bologna, Italy**	**2012**	**adult, whole**	**95% ethanol**	Qiagen
**29**	**DQ34**	**+**	**21.66**	**73**	***D. suzukii***	**Bologna, Italy**	**2012**	**adult, whole**	**95% ethanol**	Qiagen
30	DQ35	−	N/A	N/A	*D. pseudoobscura*	New Brighton, NZ	2012	adult, whole	95% ethanol	Qiagen
31	DQ36	−	N/A	N/A	*D. pseudoobscura*	New Brighton, NZ	2012	adult, whole	95% ethanol	Qiagen
32	DQ37	−	N/A	N/A	*D. hydei*	New Brighton, NZ	2012	adult, whole	95% ethanol	Qiagen
33	DQ38	−	N/A	N/A	*D. hydei*	New Brighton, NZ	2012	adult, whole	95% ethanol	Qiagen
34	DQ39	−	N/A	N/A	*D. busckii*	New Brighton, NZ	2012	adult, whole	95% ethanol	Qiagen
35	DQ40	−	N/A	N/A	*D. simulans*	New Brighton, NZ	2012	adult, whole	95% ethanol	Qiagen
36	DQ41	−	N/A	N/A	*D. pseudoobscura*	New Brighton, NZ	2012	adult, whole	95% ethanol	Qiagen
37	DQ42	−	N/A	N/A	*D. pseudoobscura*	New Brighton, NZ	2012	adult, whole	95% ethanol	Qiagen
38	DQ43	−	N/A	N/A	*D. melanogaster*	Thailand	2013	adult, whole	95% ethanol	Qiagen
39	DQ44	−	29.695	72.6	*D. subpulchrella*	China	2012	adult, whole	95% ethanol	Qiagen
40	DQ45	−	29.39	72.6	*D. subpulchrella*	China	2012	adult, whole	95% ethanol	Qiagen
41	DQ46	−	28.54	72.6	*D. subpulchrella*	China	2012	adult, whole	95% ethanol	Qiagen
42	DQ47	−	N/A	N/A	*D. subpulchrella*	China	2012	adult, whole	95% ethanol	Qiagen
43	DQ48	−	29.715	72.6	*D. subpulchrella*	China	2012	adult, whole	95% ethanol	Qiagen
44	DQ49	−	28.825	72.6	*D. subpulchrella*	China	2012	adult, whole	95% ethanol	Qiagen
**45**	**DQ50**	−	**22.02**	**73**	***D. suzukii***	**Oregon, USA**	**2012**	adult, whole	**RNALater**	Qiagen
**46**	**DQ51**	−	**21.5**	**73**	***D. suzukii***	**Oregon, USA**	**2012**	adult, whole	**RNALater**	Qiagen
**47**	**DQ52**	−	**21.175**	**73**	***D. suzukii***	**Oregon, USA**	**2012**	adult, whole	**RNALater**	Qiagen
**48**	**DQ53**	−	**22.255**	**73**	***D. suzukii***	**Oregon, USA**	**2012**	adult, whole	**RNALater**	Qiagen
**49**	**DQ54**	−	**20.765**	**73.2**	***D. suzukii***	**Oregon, USA**	**2012**	adult, whole	**RNALater**	Qiagen
**50**	**DQ55**	−	**20.66**	**73.2**	***D. suzukii***	**Oregon, USA**	**2012**	adult, whole	**RNALater**	Qiagen
51	DQ56	−	N/A	N/A	*D. repleta*	Auckland, NZ	2009	adult, whole	dry	Qiagen
52	DQ57	−	N/A	N/A	*D. neozealandica*	Whangarei, NZ	1997	larva, whole	95% ethanol	Qiagen
53	DQ60	−	N/A	N/A	*D. kirki*	Oratia, NZ	1987	adult, whole	95% ethanol	Qiagen
54	DD6	−	N/A	N/A	*D. subpulchrella*	Kanagawa, Japan	2013	adult, whole	95% ethanol	PrepGem
55	DD7	−	N/A	N/A	*D. subpulchrella*	Kanagawa, Japan	2013	adult, whole	95% ethanol	PrepGem
56	DD8	−	N/A	N/A	*D. subpulchrella*	Kanagawa, Japan	2013	adult, whole	95% ethanol	PrepGem
57	DD9	−	N/A	N/A	*D. subpulchrella*	Kanagawa, Japan	2013	adult, whole	95% ethanol	PrepGem
58	**DN23**	**+**	**22.50**	**72.8**	***D. suzukii***	**Hachijo Is, Tokyo, Japan**	**2012**	**adult, whole**	**95% ethanol**	**PrepGem**
59	DN24	−	N/A	N/A	*D. biarmipes*	Mysore, India	**2012**	adult, whole	95% ethanol	PrepGem
60	DN25	−	N/A	N/A	*D. lucipennis*	Wulai, Taiwan	**2012**	adult, whole	95% ethanol	PrepGem
61	DN26	−	N/A	N/A	*D. mimetica*	Kuala Belalong, Brunei	**2012**	adult, whole	95% ethanol	PrepGem
62	DN27	−	29.3	72.6	*D. subpulchrella*	China	**2012**	adult, whole	95% ethanol	PrepGem
63	DN28	−	N/A	N/A	*D. takahashii*	Yun Shui, Taiwan	**2012**	adult, whole	95% ethanol	PrepGem
64	DN29	−	N/A	N/A	*D. lutescens*	Mito, Honshu, Japan	**2012**	adult, whole	95% ethanol	PrepGem
65	DN30	−	N/A	N/A	*D. eugracilis*	Kuala Belalong, Brunei	**2012**	adult, whole	95% ethanol	PrepGem

All samples were run in duplicate, therefore mean *Cq* and mean Melt Peak Temperatures are provided. Positive identifications depend on the amplification as well as melt peak temperatures, with *D. suzukii* melt peaks = 73°C (−0.2/+0.4°C). Further confirmation of the identifications was obtained via analysis of the difference plot of the melt curves.

1 All species were first identified morphologically by Dr Disna Gunawardana (or Dr Gunawardana confirmed identifications by the submitters), and subsequently confirmed also via sequencing the COI gene. See footnote of Table 2 for further details on identifiers/submitters for the samples.

§ Amplification after the cut-off *Cq* value.

#### High resolution melt peak specificity

**Figure 3:**
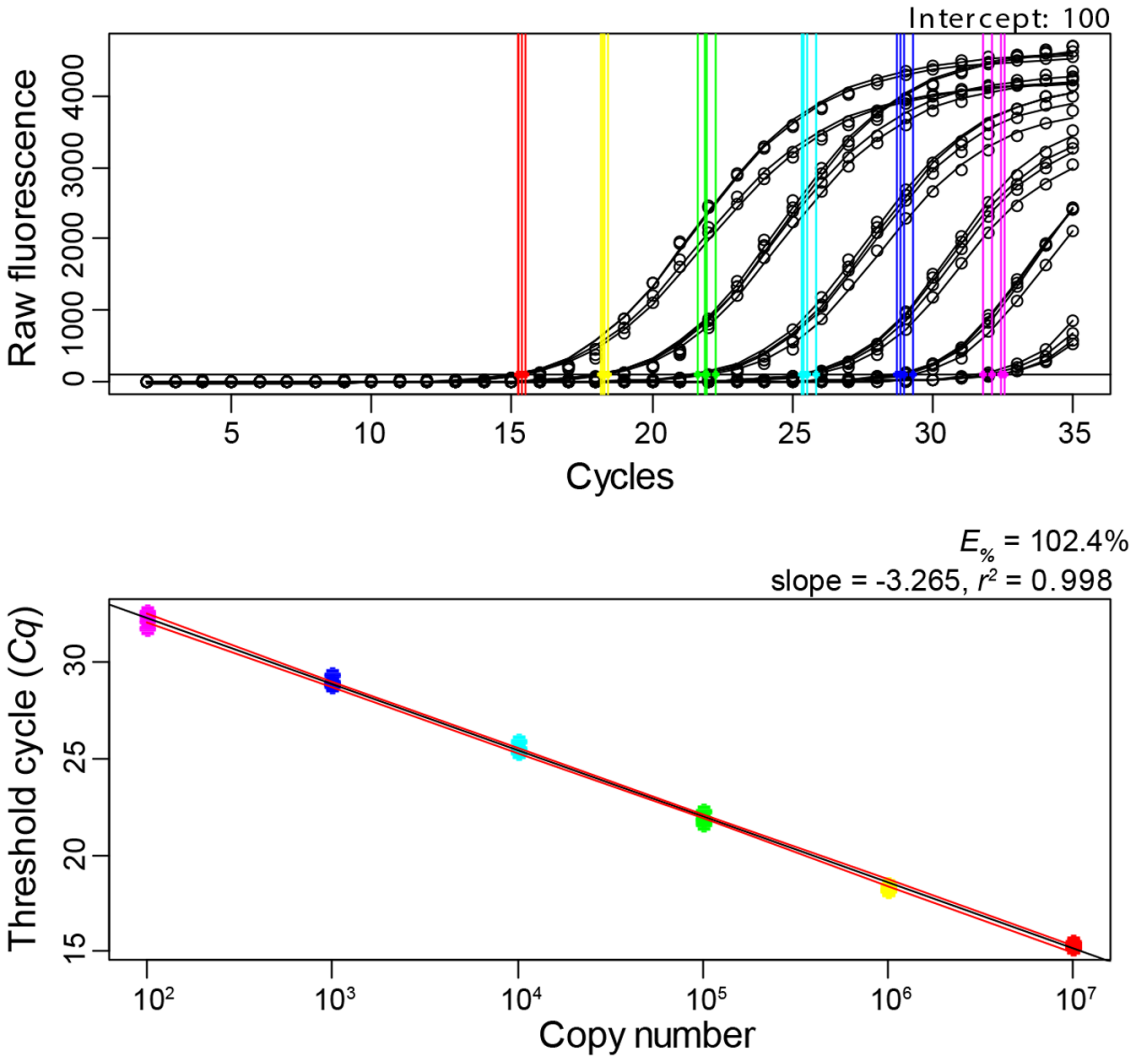
Efficiency of the real-time PCR assay for the identification of *D. suzukii*. Plasmid dilution series were used to create calibration curves for efficiency calculations. Top panel shows the amplification curves, threshold = 100 raw fluorescence units (rfu) and coloured lines indicate the *Cq* (rfu threshold cycle) values for each of the dilution series replicates. Bottom panel shows the standard curve built from Cq (threshold cycle) values against the log copy number (range = 10^7^−10^2^ copies). The 95% confidence interval of the slope is plotted in red lines and the *r^2^* = 0.998. The fit of the slope was also optimised using the Akaike Information Criteria (AIC) and the AIC statistic = 9.52.

All the 23 *D. suzukii* samples showed melt peaks in the acceptable range of 73.0±0.2°C and were clearly distinguished from *D. subpulchrella* (72.4°C±0.2°C) and the single *D. biarmipes* melt peak at 75.6°C (Figure 3a). No other melt peaks were reported.

#### High resolution melt curve analysis

The melt profiles of *D. suzukii* and *D. subpulchrella* were also compared by using the “melt study” interface of the Precision Melt Analysis software (BioRad). This provides automation for the separation of a large number of samples of *D. suzukii* and *D. subpulchrella*. It requires a dataset of melt profiles from known samples of both the species, and creates species clusters according to melt temperature and difference plot shape.

Overall, the analytical specificity of the assay including the HRM component in detecting the *D. suzukii* samples was 100% and the diagnostic specificity for the same in resulting negative for the non-target species was also 100%.

### Analytical sensitivity and performance of the real-time assay for the detection of *D. suzukii*

#### PCR amplification efficiency

The slope of the calibration curve was −3.265 (95% CI = −3.344, −3.181). The PCR efficiency was calculated at 102.4%, which is within the 95–105% range generally accepted for an efficient PCR reaction (Figure 3).

#### Linear Dynamic Range

The linear dynamic range of the calibration curve extended from 10^7^−10^2^ copies of template DNA. This linear dynamic range covered the interval of realistic target diagnostic samples, as 10^−1^−10^−2^ times diluted DNA extracts were detected within this range. Very low *Cq* variation was observed at the higher end of target detection (10^7^−10^4^ copies), while slightly higher *Cq* variation was observed for the lower end of target detection (10^3^−10^2^ copies). The correlation coefficient, *r^2^*, of the calibration curve was 0.998. The 95% confidence limits of the linear dynamic range are plotted in Figure 3.

#### Limit of Detection (LOD)

The LOD for the real-time assay was determined to be 10^2^ copies of target DNA. The calibration curve shown in Figure 3 as well as other calibration curves run (n = 3), were each able to detect all the samples (and replicates) at the 10^2^ copies/μL concentration, therefore giving 100% confidence to the LOD of 10^2^ copies.

#### Repeatability and reproducibility

Very low %CV was observed for each of the samples tested within individual runs suggesting reliable assay repeatability (Table 4). Across runs conducted at two facilities, very low %CV was reported, suggesting high assay reproducibility (Table 5).

**Table 4:** Repeatability: percentage coefficient of variation (%CV) for intrarun repeatability experiments.

Sample ID	DNA conc. (ng/μL)	%CV			
		Run 1	Run 2	Run 3	Run 4
**DQ11**	20.03	1	0.66	1.29	0.72
**DQ19**	21.25	0.25	0.47	0.4	0.38
**DQ20**	23.83	0.71	0.21	0.94	0.83
**DQ23**	25.83	0.87	1.81	0.55	0.25

Three replicates of each sample were used. Two experiments were conducted internally at PHEL (Runs 1 & 2) and two experiments externally at AHL (Runs 3 & 4). All experiments were identical and included the same four samples.

**Table 5:** Reproducibility: percentage coefficient of variation (%CV) for interrun repeatability experiments.

Sample ID	Mean *Cq*	Standard deviation	% CV
**DQ11**	20.21	0.28	1.37
**DQ19**	20.50	0.32	1.54
**DQ20**	20.10	0.18	0.91
**DQ23**	20.08	0.27	1.34

Three replicates of each sample were used. Four experiments, two conducted internally (at PHEL) and two externally (at AHL) were used to calculate %CV. All experiments were identical and included the same four samples.

### Robustness of the real-time PCR assay for *D. suzukii*: amplification of degraded/low purity/low copy number samples

Of the 14 samples tested (comprising of old samples, eggs, legs and dried material), 13 samples amplified successfully (*Cq*<29) with correct melting peaks (Table S2). The robustness of this assay, calculated as percentage of correctly identified low quality samples, was 92.8% (13/14). These samples performed poorly otherwise with conventional PCR where only a single extract (sample ID: DN15, 3 legs) weakly amplified, yielding a success rate of 7%.

### Validation of the real-time PCR assay for *D. suzukii*: Blind panel

Of the 23 samples of unknown identity, four were accurately identified as *D. suzukii*, through successful amplification and correct melt peak temperature (Table 6). No other samples showed positive amplification. The test results were independently matched to the original identities of the samples by Dr Disna Gunawardana. Independent external validation by the AHL using the blind panel assay returned identical results.

**Table 6:** Validation of the real-time PCR assay for the detection of *D. suzukii*: Blind panel results.

S.no.	Sample ID	Real-time assay			Morph ID^1^	Collection location^2^	Collection year	Life stage, body part (no. of individuals)	Host (if known)	Preservation	External validation result
		Result	Mean Cq	Mean Melt Peak (°C)							
1	D1	−	N/A	N/A	*D. immigrans*	Australia*	2007	Adult, whole (1)	Honeydew melon	95% alc.	−
2	D2	−	N/A	N/A	*D. neozealandica*	Whangarei, NLD, NZ	1990	Adult, whole (1)	bulbs	95% alc.	−
**3**	**D3**	**+**	**27.48**	**73**	***D. suzukii***	**Oregon, USA**	**2012**	**Larva, whole (1)**	**N/A**	**RNALater**	**+**
4	D4	−	N/A	N/A	*D. simulans*	Mt. Roskill, AKL, NZ	2005	Adult, leg (1)	N/A	95% alc.	−
5	D5	−	N/A	N/A	*D. immigrans*	Australia*	2011	Adult, whole (1)	Rock melon	95% alc.	−
6	D6	−	N/A	N/A	*D. immigrans*	Whangarei, NLD, NZ	1982	Adult, whole (1)	Rape	95% alc.	−
7	D7	−	N/A	N/A	*D. immigrans*	Auckland, NZ	1971	Larva, whole (1)	N/A	95% alc.	−
8	D8	−	N/A	N/A	*D. immigrans*	Auckland, NZ	1971	Pupa, whole (1)	N/A	95% alc.	−
**9**	**D9**	**+**	**25.005**	**73.4**	***D. suzukii***	USA, ex: Contra Costa*	**2010**	**Adult, whole (1)**	**N/A**	**95% alc.**	**+**
10	D10	−	N/A	N/A	*Sapromyza* spp.	Tauranga, BP, NZ	1995	Larva, posterior end (1)	N/A	95% alc.	−
11	D11	−	N/A	N/A	*D. busckii*	Australia*	2007	Adult, leg (1)	N/A	95% alc.	−
12	D12	−	N/A	N/A	*D. repleta*	New Zealand	2009	Adult, leg (1)	N/A	95% alc.	−
13	D13	−	N/A	N/A	*D. immigrans*	Ecuador*	2007	Adult, leg (1)	Pineapple	95% alc.	−
**14**	**D14**	**+**	**23.735**	**73**	***D. suzukii***	**Bologna, Italy**	**2013**	**Adult, whole (1)**	**Plum**	**95% alc.**	**+**
15	D15	−	N/A	N/A	*D. pseudoobscura*	New Brighton, MC, NZ	2012	Adult, whole (1)	N/A	95% alc.	−
16	D16	−	N/A	N/A	*D. hydei*	New Brighton, MC, NZ	2012	Adult, leg (1)	N/A	95% alc.	−
17	D17	−	N/A	N/A	*D. pseudoobscura*	New Brighton, MC, NZ	2012	Larva, whole (1)	N/A	95% alc.	−
18	D18	−	N/A	N/A	*D. pseudoobscura*	New Brighton, MC, NZ	2012	Pupa, whole (1)	N/A	95% alc.	−
**19**	**D19**	**+**	**22.17**	**73.2**	***D. suzukii***	**Oregon, USA**	**2012**	**Adult, leg (1)**	**N/A**	**RNALater**	**+**
20	D20	−	N/A	N/A	*D. melanogaster*	Australia*	2006	Adult, whole (1)	Rock melon	dry	−
21	D21	−	N/A	N/A	*D. kirkii*	Oratia, AKL, NZ	1987	Adult, leg (1)	N/A	95% alc.	−
22	D22	−	N/A	N/A	*D. melanogaster*	Auckland, NZ	1970	Adult, whole (1)	N/A	95% alc.	−
23	D23	−	N/A	N/A	*D. melanogaster*	USA*	2011	Adult, whole (1)	Mandarin	95% alc.	−

The samples returning a positive result for *D. suzukii* are in bold. All samples were tested in duplicates.

1 Morphological IDs were carried out by Dr. Disna Gunawardana and were corroborated with assay results by her independently.

2 Collection locations with “*” against them denote that the country of origin are provided instead because these were samples intercepted at the New Zealand border. For New Zealand (NZ) locations, Crosby codes have been used to denote the area codes.

## Discussion

Real-time PCR-based techniques are increasingly being used for the accurate and rapid identification of pest species throughout the world. Plant pathogens such as fungi, bacteria and viruses provide excellent examples of pests routinely identified using real-time PCR [28], [38]–[40]. Insects form a large and diverse group of plant pests, but few insects have species-specific real-time PCR assays. These assays are available for economically significant and difficult to identify pests such as *Thrips palmi* [41] and *Bactrocera latifrons* [42]. Both these species cause significant damage and are often intercepted as immature stages making morphological identification difficult, and in some cases impossible. Similarly, *Drosophila suzukii*, which is an emerging pest of high economic significance, is also difficult to identify as a larva. In fact, its first record from California, United States was originally a misidentification [5]. This mistake went undiscovered until 2009 when numerous reports of massive infestations of drosophilid larvae surfaced from many parts of California [5]. By this time the species had spread into over 20 counties within California, as well as into Oregon, Washington, Florida and British Columbia (Canada) [5]. This example highlights the need for an accurate method of identification of *D. suzukii* larvae.

In this study, we have developed a novel real-time PCR assay for the detection and identification of *D. suzukii*. Using real-time PCR eliminates post-PCR processing, reducing the time to identification by several hours. EvaGreen provides high specificity [43], can be transferred to any laboratory, and run cheaply as it does not require expensive fluorescently labelled probes.

The assay was tested thoroughly *in silico* during the development stage and experimentally during the specificity testing stage. We tested a total of 17 *Drosophila* species in the specificity testing of this assay (Table 1). Despite a cross continent procurement effort, some elusive species from the *D. suzukii* species subgroup were not available for testing and their sequences were not available on GenBank either. Of these, *D. pulchrella* (China, Japan and India) and *D. oshimai* (Japan) are the only species of concern, due to their distributions overlapping with that of *D. suzukii*. Additionally, *D. prolongata, D. immacularis* and *D. tristipennis* that are found in India and Japan may pose an increased risk, if their distribution increases northward or are transported northward on trade routes. The remaining species, such as *D. apodemata, D. ashburneri*, *D. hypomelana, D. plagiata* and *D. unipunctata*, are of limited concern in influencing the success of this assay as these species are either obscure, or have very limited or very characteristic distributions, not overlapping that of *D. suzukii*. One New Zealand species that remains untested in any form is *D. brouni* but it is only remotely related to *D. suzukii*.

The amplification by the primer pair although biased towards the target species, narrows down the identification to a couple of closely related species. The high resolution melt peaks further differentiate between *D. suzukii* and close congener *D. subpulchrella* accurately. *Drosophila subpulchrella* also causes crop damage but due to its limited distribution in parts of China and Japan [5],[44] it is not considered a pest of concern. It is unlikely to be encountered via major trade routes such as USA and Europe that have high risk to transport *D. suzukii* [5]. Nonetheless, we can easily distinguish this species from *D. suzukii* through melting profile differences of the amplicons produced in this assay. Should *D. subpulchrella* become a pest of concern, our assay could be easily used for the sensitive and accurate identification of this species as well.

The HRM assay performed optimally with 100% diagnostic and analytical specificity in samples derived from legs of adults, whole larvae and whole adults (extracted using Qiagen protocol). Such accuracy is ideal in a diagnostic framework. The assay also exhibited high efficiency and sensitivity, with the ability to identify reliably as low as 100 copies of the template DNA. Three calibration curves with replicates were used to validate the limit of detection. Furthermore, the low %CV observed within and across runs conducted at two different testing facilities indicates low intra-assay and inter-assay variance. Such consistency is ideal for a diagnostic assay, with minimal operator, handling and instrument differences observed between laboratories.

The high sensitivity of this assay means that samples yielding low copy numbers can also be analysed. Such is often the case when extracting DNA from intercepted eggs, or the whole specimen cannot be used. This HRM assay accurately identified *D. suzukii* from single or multiple egg samples that could not have been confidently identified to species level morphologically. Additionally, *D. suzukii* was successfully identified from low quality samples extracted with a crude DNA preparation method. This crude DNA extraction method (prepGem) is rapid, reducing the time for the identification by several hours compared to extracting high quality DNA using the Qiagen protocol. Although 100% success with degraded samples was not observed, if was markedly higher than using conventional PCR amplification.

The application of a diagnostic assay within the New Zealand quarantine framework is characterised by three main tenets: high specificity, high sensitivity and swift results. This HRM assay fulfils each of these criteria. The final validation, both internally and independently at an external testing facility (AHL) using a blind panel, provides further confidence that this assay can be reliably used in a routine diagnostic framework in New Zealand and overseas. It is important to note that border-intercepted organisms were included in the panel to simulate a realistic situation, and it included a *D. suzukii* specimen actually intercepted at the New Zealand border in 2010. It is important to note that this assay is already being employed at the PHEL quarantine testing facility to investigate suspect spotted-wing drosophila interceptions.

In conclusion, the novel real-time PCR developed here is suitable for routine use by diagnostic and research agencies, for facilitating exports and imports, as well as in aiding border security agencies worldwide to limit and monitor the spread of this pest. This assay provides a rapid, accurate and specific alternative to morphology or barcoding methods of identification for *D. suzukii*. Since real-time PCR machines are available in 96-well or 384-well format, and semi-automated extraction methods are becoming more available, this method is amenable to high throughput applications, often necessary during large scale surveys for delimiting infestation during an incursion. We have fully optimised this assay for immediate deployment in New Zealand. We suggest pre-deployment testing in locations outside New Zealand, to ensure no false positives are detected although they would be highly unlikely.

## Supporting Information

Table S1 **Accession numbers of the sequences of the samples used in the design phase and the specificity-testing phase of the assay development.**
(XLSX)

Table S2 **Robustness of the real-time PCR assay for the detection of** ***D. suzukii*** **.** All low quality and low DNA samples were run in duplicate, therefore mean *Cq* and mean Melt Peak Temperatures are provided. Positive identifications depend on the amplification as well as melt peak temperatures, with *D. suzukii* melt peaks = 73°C (−0.2/+0.4°C).
(XLSX)
